# Screen time, social media use, and weight-related bullying victimization: Findings from an international sample of adolescents

**DOI:** 10.1371/journal.pone.0299830

**Published:** 2024-04-17

**Authors:** Kyle T. Ganson, Nelson Pang, Jason M. Nagata, Catrin Pedder Jones, Faye Mishna, Alexander Testa, Dylan B. Jackson, David Hammond

**Affiliations:** 1 Factor-Inwentash Faculty of Social Work, University of Toronto, Toronto, Ontario, Canada; 2 Department of Pediatrics, University of California, San Francisco, California, United States of America; 3 MRC Epidemiology Unit, School of Clinical Medicine, University of Cambridge, Cambridge, United Kingdom; 4 Department of Management, Policy and Community Health, University of Texas Health Science Center at Houston, Houston, TX, United States of America; 5 Department of Population, Family, and Reproductive Health, Johns Hopkins Bloomberg School of Public Health, Baltimore, MD, United States of America; 6 School of Public Health and Health Systems, University of Waterloo, Waterloo, Ontario, Canada; Florida Atlantic University Charles E Schmidt College of Medicine, UNITED STATES

## Abstract

Screen time, social media, and weight-related bullying are ubiquitous among adolescents. However, little research has been conducted among international samples. Therefore, the aim of this study was to determine the association between screen time, social media use, and weight-related bullying victimization among an international sample of adolescents from six countries. Data from the 2020 International Food Policy Study Youth Survey were analyzed (N = 12,031). Multiple modified Poisson regression models were estimated to determine the associations between weekday hours of five forms of screen time, and total screen time, and use of six contemporary social media platforms and weight-related bullying victimization. Analyses were conducted among the overall sample, and stratified by country (Australia, Canada, Chile, Mexico, United Kingdom, United States). Greater hours of weekday screen time and use of each of the six social media platforms were associated with weight-related bullying victimization among the sample. Each additional hour of social media use was equivalent to a 13% (confidence interval [CI] 1.10–1.16) increase in the prevalence of weight-related bullying victimization. The use of Twitter was associated with a 69% (CI 1.53–1.84) increase in the prevalence of weight-related bullying victimization. Associations between hours of weekday screen time, use of six social media, and weight-related bullying victimization differed by country. Findings underscore the associations between screen time, social media, and weight-related bullying among a sample of adolescents from six medium- and high-income countries. Country-specific and global public health and technology efforts are needed to address this burgeoning social problem.

## Introduction

Screen time and social media use are common among adolescents [1], and time spent engaging with screens and social media has increased during the COVID-19 pandemic [2]. For many adolescents screen time and social media use are forms of social connection and entertainment. Surveys suggest that social media and screen use is rapidly growing with estimates of 88%-99% in Australia, Canada, the UK, and the USA [3–6]. Additionally, there are nearly 5 billion social media users worldwide [7]. These statistics underscore the pervasive global use of screens and social media.

High levels of screen time and social media use are connected with decreased physical activity [8] and greater body mass index (BMI) [9]. Despite potential social benefits [10], screen time and social media use present possible risks, such as cyberbullying [11]. Cyberbullying is particularly problematic as it can occur with relative anonymity [12]. One of the most common types of bullying is weight-related bullying victimization (referred to as weight-related bullying hereafter) [13,14]. Weight-related bullying is highly prevalent among youth around the world, including in Denmark, Australia, Canada, Iceland, and the USA, among others [15–17], and a recent meta-analysis found that children and adolescents with “obesity” had greater odds of experiencing bullying compared to their “normal” weight peers [18]. Weight-related bullying can have many adverse psychosocial effects, including weight gain, decreased involvement in physical activity, body dissatisfaction, eating disorder behaviors, low self-esteem, and depression [19–21].

The social-ecological model of cyberbullying is a theoretical model focused on youth that builds upon Bronfenbrenner’s ecological systems theory and Swearer and Espelage’s social-ecological model of bullying [22]. This model offers a comprehensive lens to better understand the intricate pathways linking screen time, social media use, and weight-related bullying with an additional focus on digital context. This theory recognizes that these phenomena are not isolated but rather influenced by a range of interconnected factors across different levels. At the individual level, screen time and social media exposure can shape personal body image perceptions and self-esteem. Likewise, sociodemographic characteristics like gender, race/ethnicity, sexual orientation, and body image can contribute to bullying perpetration and victimization [23]. Whereas interpersonal relationships can influence attitudes towards social media use, bullying, and group norms [24]. The community level, encompasses schools and local environments, creating the norms of body image, bullying, screen time, and social media use [25,26]. The societal level includes media portrayals and cultural standards, which influence how individuals perceive body ideals and engage in online activity [27,28]. Lastly, the digital context encompasses how digital media is intertwined with many domains of life and examines how types of social media may facilitate social media use and weight-related bullying [22]. The framework will help conceptualize the analysis and results.

Prior research in the United States has documented that weight-related bullying is common in online spaces [29], and it is well known that weight stigma is pervasive across media, including social media [30]. Furthermore, research among adolescents in 47 regions and countries found associations between social media use and cyberbullying [31]. To date, however, a major limitation of studies investigating screen time, social media use, and cyberbullying is the lack of focus on weight-related bullying, specifically. Additionally, most research on the association between screen time, social media use, and bullying lacks large and diverse international samples, and there is limited information on the specific screen time modalities and social media platforms. Therefore, the aim of this study was to determine the association between five forms of screen time, use of six contemporary social media platforms and weight-related bullying among a sample of adolescents from six countries.

## Methods

A secondary data analysis of the 2020 International Food Policy Study (IFPS) Youth Survey (N = 12,031) was conducted in 2022. IFPS Youth Survey is an annual repeat cross-sectional survey conducted in Australia (n = 1,595), Canada (n = 3,895), Chile (n = 1,615), Mexico (n = 1,823), the United Kingdom (n = 1,521), and the United States (n = 1,582). Data were collected via self-completed web-based surveys conducted in November-December 2020 with youth aged 10 to 17 years. Respondents were recruited through parents/guardians enrolled in the Nielsen Consumer Insights Global Panel and their partners’ panels. The total participation rate was 3.4%, with 353,443 invitations sent to potential respondents; 15,515 (4.4%) potential respondents accessed the survey link; and 12,031 (3.4%) respondents completed the IFPS survey and were retained in the sample [32]. A combination of probabilistic and non-probabilistic sampling methods was used to sample for this study. To increase representativeness, post-stratification sample weights were constructed and applied for each country separately based on known population totals by age, sex, region, and ethnicity (except in Canada). After eligibility screening, all potential respondents were provided with information about the study and asked to provide assent. The study was reviewed by and received ethics clearance through the University of Waterloo Research Ethics Committee (ORE# 41477). A full description of the study methods can be found elsewhere [32].

## Measures

### Screen time and social media use

Screen time was assessed based on number of weekday hours spent using five contemporary recreational screen modalities, including “Watching YouTube,” “On social media (including messaging, posting, or liking posts),” “Watching TV shows, series, or movies,” “Playing games on smartphones, computers, or game consoles,” and “Browsing, reading websites, Googling, etc.”. Total weekday screen time use was calculated using the sum of the five screen modalities [1].

Social media use was assessed based on the presence (1) or absence (0) of current use of six contemporary forms of social media, including “Facebook,” “Instagram,” “TikTok,” “Twitter,” “Snapchat,” and “Twitch” [33].

### Weight-related bullying

Weight-related bullying was assessed using the question, “Do you get teased or made fun of because of your weight?” Response options included, “All the time;” “A lot;” “Sometimes;” “Rarely;” and “Never.” Responses were dichotomized to never/rarely (0) and sometimes/a lot/all the time (1) to align with prior research [20,34].

### Sociodemographic variables

Sociodemographic variables included sex (girls, boys), age (continuous), BMI z-score (World Health Organization classification) [35], family income adequacy, and country. Adolescent race/ethnicity was categorized into binary majority and minority groups based on census questions asked in each country (see Table 1 footnote for description) [32].

**Table 1:** Characteristics of adolescent participants from the 2020 international food policy study (N = 12,031).

	% / M (SD)
Sex	
Female	49.0
Male	51.0
Age	13.5 (2.2)
Race/Ethnicity^a^	
Majority	73.4
Minority	26.6
BMI Z-Score Classification	
Z-Score < -3 (“Severe Thinness”)	1.8
-3 ≤ Z-Score < -2 (“Thinness”)	1.8
-2 ≤ Z-Score ≤ 1 (“Normal Weight”)	43.2
1 < Z-Score ≤ 2 (“Overweight”)	17.1
Z-Score > 2 (“Obesity”)	11.4
Missing	24.7
Family Income Adequacy	
Very Easy	7.8
Easy	20.0
Neither Easy nor Difficult	38.2
Difficult	26.0
Very Difficult	7.3
Don’t Know	0.6
Country	
Canada	32.4
Australia	13.3
United Kingdom	12.6
United States	13.2
Mexico	15.1
Chile	13.4
Weight-Related Bullying	
Never/Rarely	83.5
Sometimes/A Lot/All the Time	16.5
Screen Time, Hours per Weekday	
YouTube Hours	1.7 (1.4)
Social Media Hours	1.4 (1.4)
TV Hours	1.7 (1.3)
Video Game Hours	1.7 (1.5)
Browsing Web Hours	1.1 (1.2)
Total Screen Time Hours	7.5 (5.0)
Social Media Platform Use (“Yes” Responses)	
Facebook	49.6
Instagram	55.0
TikTok	48.5
Twitter	21.7
Snapchat	38.4
Twitch	12.4

Note: Preconstructed sample weighting applied to all analyses.

M = Mean; SD = Standard deviation; BMI = Body mass index.

^a^Canada: Majority if ‘White (European descent)’ is only category checked or ‘other’ response such as Caucasian, Canadian, Jewish; minority if any other category checked; Australia: Majority if only speak English at home; minority if speak a language other than English at home, or indicated they are aboriginal or Torres Straight Islander; United Kingdom: Majority if only checked a ‘white’ option; minority if checked any other category; United States: Majority if ‘white’ is only category checked; minority if any other category checked; Mexico: Majority if do not consider self indigenous; minority if consider self indigenous; Chile: Majority if do not consider self indigenous; minority if consider self indigenous.

### Statistical analyses

Multiple modified Poisson regression models (12 in total) were estimated to determine the association between screen time, social media use, and weight-related bullying, with coefficients transformed to prevalence ratios [36], adjusting for the sociodemographic variables. Adjusted analyses were conducted among the overall sample and stratified by country. We also tested for effect modification by sex, BMI z-score, and race/ethnicity. Data were weighted with post-stratification sample weights constructed using a raking algorithm with population estimates from the census in each country based on age group, sex, region, and ethnicity (except in Canada) [32]. Statistical significance was defined as two-sided p < 0.05. Statistical analyses were conducted using Stata version 17 [37].

## Results

Among the sample, nearly 17% reported weight-related bullying sometimes, a lot, or all the time. Mean total screen time per weekday was 7.5 hours (SD = 5.0), with Instagram reported to be the most frequently used social media platform (55%; Table 1). See S1 Table for sample characteristics across countries.

Results showed significant associations between all screen time and social media use items and weight-related bullying among the overall sample (Table 2). Greater hours of social media use (prevalence ratio [PR] 1.13, 95% confidence interval [CI] 1.10–1.16) and browsing the web (PR 1.13, 95% CI 1.10–1.17) were most strongly associated with weight-related bullying. Use of Twitter (PR 1.69, 95% CI 1.53–1.84) was most strongly associated with weight-related bullying among the social media platforms.

**Table 2:** Associations between screen time and social media platform use and weight-related bullying among adolescent participants from the 2020 international food policy study (N = 12,031).

**Screen Time, Hours per Weekday **	PR (95% CI)^a^	p
YouTube Hours	1.08 (1.04–1.11)*	< 0.001
Social Media Hours	1.13 (1.10–1.16)*	< 0.001
TV Hours	1.05 (1.02–1.08)*	0.002
Video Game Hours	1.09 (1.06–1.12)*	< 0.001
Browsing Web Hours	1.13 (1.10–1.17)*	< 0.001
Total Screen Time Hours	1.03 (1.03–1.04)*	< 0.001
**Social Media Platform Use **	PR (95% CI)^a^	p
Facebook	1.39 (1.26–1.53)*	< 0.001
Instagram	1.35 (1.22–1.48)*	< 0.001
TikTok	1.26 (1.15–1.37)*	< 0.001
Twitter	1.69 (1.53–1.84)*	< 0.001
Snapchat	1.25 (1.14–1.37)*	< 0.001
Twitch	1.49 (1.34–1.66)*	< 0.001

Note: Each cell represents the abbreviated outputs of 12 modified Poisson regression models with screen time and social media platform use as the independent variables and weight-related bullying as the dependent variable. Preconstructed sample weighting applied to all analyses.

* indicates statistical significance (p < 0.05).

PR = Prevalence ratio; CI = Confidence interval.

^a^Adjusted for age, sex, race/ethnicity, body mass index z-score classification, family income adequacy, and country.

Result from stratified analyses (S2–S7 Tables) showed differing patterns of association between screen time, social media use, and weight-related bullying across countries. Specifically, associations were strongest in Canada, Australia, and the United Kingdom, while there were fewer significant associations in the United States, Mexico, and Chile. Additionally, we tested for effect modification by sex, BMI z-score, and race/ethnicity. There were no significant interactions with BMI z-score or race/ethnicity. However, sex modified the relationship between time spent playing video games and weight-related bullying, whereby males, compared to females, were less likely to experience weight-related bullying as time playing video games increased (95% CI 0.88–0.98, p = 0.013). Additionally, sex modified the relationship between use of Twitch and weight-related bullying, whereby males, compared to females, were less likely to experience weight-related bullying if they reported using Twitch (95% CI 0.60–0.91, p = 0.006).

## Discussion

Findings from the study report relationships between increased screen time hours, social media use, and weight-related bullying among an international sample of adolescents. Our study adds to the literature on screen time among adolescents by including an international sample of participants from six middle- and high-income countries, and by investigating multiple contemporary forms of screen time and social media. The prevalence of weight-related bullying significantly increased as weekday hours of screen time also increased, including a 13% increase in prevalence for each additional hour of social media use and browsing the web. Similarly, the prevalence of weight-related bullying was significantly higher with use of all six social media platforms, with the prevalence of weight-related bullying increasing 69% with Twitter use. Importantly, these finding are independent of several potential confounding factors, including participant country, underscoring the global relevance of our findings. These findings are important given the known connections between screen time, social media use, weight-related bullying, and body dissatisfaction and eating disorders [20,21,38,39].

While not specifically tested within this study, the findings may be explained by multiple mechanisms. First, greater time spent on screens and social media may increase the exposure of adolescents to a high degree of weight stigma, which is pervasive across media formats [30]. Secondly, extended time on screens and social media may increase weight due to the displacement of physical activity [40], which in turn may impact body satisfaction and increase possible the likelihood of weight-related bullying. Third, it is possible that greater time on screens and social media can shape attitudes due to social and group norms [24], thus increasing the risk of weight-related bullying. Fourth, cultural body ideals and standards are perpetuated in various media, including social media, which may influence online activity [27,28], including weight-related bullying. Future research is needed to determine the mechanisms underlying the association between screen time, social media use, and weight-related bullying among adolescents.

Several unique findings should be highlighted. First, it is notable that hours playing video games and use of the social media platform, Twitch, had strong associations with weight-related bullying. Twitch is commonly used by young people to live stream themselves playing video games, and prior research has reported high levels of bullying and harassment on this platform during live streams [41]. Given the live streaming nature of this platform, it may be difficult for individuals to hide their body to viewers, opening an avenue for weight-related bullying. Furthermore, females, compared to males, appear more at-risk for experiencing weight-related bullying when playing video games and using Twitch. This finding may be the result of online video games, and platforms such as Twitch, being spaces of significant sexism towards females and toxicity [42–44]. Second, use of Twitter, a predominately text-based platform, had the strongest association with weight-related bullying of all social media platforms. This aligns with research reporting high levels of weight stigma on Twitter [45]. Additionally, it may be that adolescents who are more susceptible to weight-related bullying avoid primarily image-based platforms, such as Instagram. Both findings should be emphasized given they are discordant with the common media and public narrative that Facebook and Instagram are the preeminent problematic spaces of social engagement for young people [39,46].

Findings also showed differing patterns of association across countries. These findings are important to consider given they may implicate the role of screen time and social media use in relation to weight-related bullying. For example, given fewer significant findings in Chile and Mexico, it may be that adolescents in these middle- and lower-high-income countries have less access to screens and social media compared to higher income countries, such as Canada and Australia. Interestingly, however, there were fewer significant findings in the United States compared to Canada, Australia, and the United Kingdom, which is surprising given the pervasiveness of screen use among American adolescents [1,2,33].

There are several limitations to be noted. The participants were recruited via a non-probability sampling, which may reduce the external validity of the findings. The items were based on self-report, which may have increased the risk of reporting and recall bias, particularly regarding estimating hours of screen time. The data are cross-sectional, precluding causal interpretations. Additionally, the screen time and social media use items did not inquire about the quality or content of use, which emphasizes the need for future research. While based on prior research, there was no specific assessment of the validity and reliability of the items in this study. Despite adjusting for several relevant confounders (i.e., age, race/ethnicity, body mass index z-score classification, family income adequacy, and country), there is also the potential for unmeasured confounders that may impact the associations found in this study. Thus, future research is needed to corroborate these findings. Finally, the weight-related bullying item did not indicate a specific context, whereby participants may also be experiencing weight-related bullying aside from the online space. However, data was collected during the COVID-19 pandemic when lockdowns and closures moved many social interactions virtually. Given that data were collected during the COVID-19 pandemic, it would be relevant to investigate these associations among a longitudinal cohort study to assess the impacts of the COVID-19 pandemic on screen time, social media use, and weight-related bullying. Furthermore, we were unable to assess the role of weight-related bullying perpetration on victimization, which may be a relevant area of future investigation given the overlap between perpetration and victimization of cyberbullying [47,48]. Strengths of the study include the international sample and use of several unique and contemporary screen time and social media use items.

Considering these strengths and limitations, there are important implications from the study findings. First, social media companies should be aware of the potential for bullying and harassment that occur on their platforms, particularly among adolescents, and adjust user policies and algorithms accordingly. This is particularly important given the international reach and global impact of social media companies and their technologies, including an estimated 5 billion users worldwide [7]. Second, country-specific clinical and public health prevention and intervention efforts can be improved to provide practitioners, parents, and adolescents with specific means to monitor, control, and reduce screen time, social media use, and weight-related bullying. Indeed, organizations like the American Psychological Association emphasize the need for minimizing harm to adolescents for being exposed to “cyberhate” (i.e., cyberbullying), specifically related to one’s individual characteristics (i.e., weight) [49]. Third, given the pervasiveness of weight stigma and weight-related bullying in media, including social media [29,30], it is highly relevant to increase social media literacy among adolescents to reduce exposure to, and protect against, weight-related bullying. This may include specific training to recognize and critique weight-related bullying online.

## Conclusion

Findings from this study describe the association between screen time, social media use, and weight-related bullying among an international sample of adolescents. Notably, greater time spent on screens, and use of six contemporary forms of social media (e.g., Instagram, Twitch), were associated with a greater prevalence of experiencing weight-related bullying.

## Supporting information

S1 Table Characteristics of adolescent participants from the 2020 international food policy study stratified by country (N = 12,031). (DOCX)

S2 Table Associations between screen time and social media platform use and weight related bullying among adolescent participants in Canada from the 2020 international food policy study (n = 3,895). (DOCX)

S3 Table Associations between screen time and social media platform use and weight-related bullying among adolescent participants in Australia from the 2020 international food policy study (n = 1,595). (DOCX)

S4 Table Associations between screen time and social media platform use and weight-related bullying among adolescent participants in the United Kingdom from the 2020 international food policy study (n = 1,521). (DOCX)

S5 Table Associations between Screen time and social media platform use and weight-related bullying among adolescent participants in the United States from the 2020 international food policy study (n = 1,582). (DOCX)

S6 Table Associations between screen time and social media platform use and weight-related bullying among adolescent participants in Mexico from the 2020 international food policy study (n = 1,823). (DOCX)

S7 Table Associations between screen time and social media platform use and weight-related bullying among adolescent participants in Chile from the 2020 international food policy study (n = 1,615). (DOCX)
