# Supplementary material for: Screen time, social media use, and weight-related bullying victimization: Findings from an international sample of adolescents
Source: PLoS One. 2024 Apr 17;19(4):e0299830. doi: 10.1371/journal.pone.0299830 (PMC11023391; doi:10.1371/journal.pone.0299830)
Supplement: S1 Table — (DOCX) [file pone.0299830.s001.docx]

| S1 Table.  Characteristics of Adolescent Participants from the 2020 International Food Policy Study Stratified by Country (N = 12,031) | | | | | | | |
| --- | --- | --- | --- | --- | --- | --- | --- |
|  | Canada | Australia | United Kingdom | United States | Mexico | Chile |  |
|  | % / M (SD) | % / M (SD) | % / M (SD) | % / M (SD) | % / M (SD) | % / M (SD) | p^a^ |
| Sex |  |  |  |  |  |  | 0.999 |
| Female | 49.1 | 48.7 | 48.7 | 49.0 | 49.3 | 48.9 |  |
| Male | 50.9 | 51.3 | 51.3 | 51.0 | 50.7 | 51.1 |  |
| Age | 13.5 (2.3) | 13.4 (2.2) | 13.5 (2.0) | 13.5 (2.2) | 13.4 (2.1) | 13.5 (2.2) | 0.722 |
| Race/Ethnicity^b^ |  |  |  |  |  |  | < 0.001 |
| Majority | 70.4 | 74.2 | 82.8 | 52.0 | 80.0 | 85.2 |  |
| Minority | 29.6 | 25.8 | 17.2 | 48.0 | 20.0 | 14.8 |  |
| BMI Z-Score Classification |  |  |  |  |  |  | < 0.001 |
| Z-Score < -3 (“Severe Thinness”) | 1.7 | 2.4 | 2.1 | 3.0 | 1.0 | 1.0 |  |
| -3 ≤ Z-Score < -2 (“Thinness”) | 2.5 | 2.3 | 2.0 | 2.0 | 0.7 | 0.8 |  |
| -2 ≤ Z-Score ≤ 1 (“Normal Weight”) | 49.9 | 41.4 | 34.5 | 45.0 | 40.9 | 37.7 |  |
| 1 < Z-Score ≤ 2 (“Overweight”) | 15.3 | 15.7 | 12.4 | 20.0 | 22.2 | 18.4 |  |
| Z-Score > 2 (“Obesity”) | 9.4 | 10.2 | 7.0 | 15.4 | 14.9 | 13.6 |  |
| Missing | 21.1 | 27.9 | 42.1 | 14.7 | 20.3 | 28.5 |  |
| Family Income Adequacy |  |  |  |  |  |  | < 0.001 |
| Very Easy | 8.2 | 11.1 | 7.1 | 14.7 | 3.9 | 2.0 |  |
| Easy | 22.2 | 23.4 | 21.6 | 25.7 | 17.2 | 7.2 |  |
| Neither Easy nor Difficult | 40.2 | 36.5 | 39.0 | 32.6 | 38.7 | 39.5 |  |
| Difficult | 23.9 | 22.4 | 25.7 | 20.2 | 29.7 | 26.4 |  |
| Very Difficult | 5.0 | 5.7 | 5.9 | 6.6 | 9.8 | 13.7 |  |
| Don’t Know | 0.5 | 0.9 | 0.7 | 0.2 | 0.6 | 1.1 |  |
| Weight-Related Bullying |  |  |  |  |  |  | < 0.001 |
| Never/Rarely | 85.7 | 76.7 | 84.5 | 81.0 | 85.8 | 83.8 |  |
| Sometimes/A Lot/All the Time | 14.3 | 23.3 | 15.5 | 19.0 | 14.2 | 16.2 |  |
| Screen Time, Hours per Weekday |  |  |  |  |  |  |  |
| YouTube Hours | 1.5 (1.4) | 1.4 (1.3) | 1.4 (1.2) | 1.7 (1.4) | 1.9 (1.4) | 2.2 (1.5) | < 0.001 |
| Social Media Hours | 1.2 (1.4) | 1.1 (1.3) | 1.2 (1.2) | 1.3 (1.3) | 1.7 (1.4) | 2.1 (1.6) | < 0.001 |
| TV Hours | 1.5 (1.3) | 1.5 (1.2) | 1.6 (1.2) | 2.0 (1.4) | 1.9 (1.3) | 1.9 (1.4) | < 0.001 |
| Video Game Hours | 1.6 (1.5) | 1.3 (1.3) | 1.5 (1.3) | 1.8 (1.4) | 1.8 (1.4) | 2.4 (1.6) | < 0.001 |
| Browsing Web Hours | 0.9 (1.1) | 0.9 (1.1) | 0.9 (1.0) | 1.1 (1.1) | 1.3 (1.2) | 1.4 (1.4) | < 0.001 |
| Total Screen Time Hours | 6.8 (4.9) | 6.2 (4.6) | 6.5 (4.3) | 7.8 (5.0) | 8.6 (4.8) | 10.0 (5.3) | < 0.001 |
| Social Media Platform Use (“Yes” Responses) |  |  |  |  |  |  |  |
| Facebook | 37.3 | 48.6 | 42.6 | 57.6 | 77.9 | 46.9 | < 0.001 |
| Instagram | 51.3 | 49.7 | 51.3 | 59.3 | 54.5 | 69.2 | < 0.001 |
| TikTok | 45.1 | 38.1 | 47.3 | 46.7 | 57.8 | 59.1 | < 0.001 |
| Twitter | 14.9 | 20.3 | 19.6 | 32.5 | 28.0 | 23.8 | < 0.001 |
| Snapchat | 44.1 | 40.2 | 48.3 | 45.6 | 29.6 | 16.0 | < 0.001 |
| Twitch | 9.6 | 8.8 | 10.4 | 16.0 | 14.7 | 18.3 | < 0.001 |
| Note: Preconstructed sample weighting applied to all analyses.  M = Mean; SD = Standard deviation; BMI = Body mass index  ^a^ Statistical differences determined using independent samples t-tests for continuous variables and chi-square test for categorial variables.  ^b^ Canada: majority if ‘White (European descent)’ is only category checked or ‘other’ response such as Caucasian, Canadian, Jewish; minority if any other category checked; Australia: majority if only speak English at home; minority if speak a language other than English at home, or indicated they are aboriginal or Torres Straight Islander; United Kingdom: majority if only checked a ‘white’ option; minority if checked any other category; United States: majority if ‘white’ is only category checked; minority if any other category checked; Mexico: majority if do not consider self indigenous; minority if consider self indigenous; Chile: majority if do not consider self indigenous; minority if consider self indigenous. | | | | | | | |
