# Supplementary material for: Screen time, social media use, and weight-related bullying victimization: Findings from an international sample of adolescents
Source: PLoS One. 2024 Apr 17;19(4):e0299830. doi: 10.1371/journal.pone.0299830 (PMC11023391; doi:10.1371/journal.pone.0299830)
Supplement: S2 Table — (DOCX) [file pone.0299830.s002.docx]

| S2 Table.  Associations between Screen Time and Social Media Platform Use and Weight-Related Bullying among Adolescent Participants in Canada from the 2020 International Food Policy Study (n = 3,895) | | |
| --- | --- | --- |
| **Screen Time, Hours per Weekday** | PR (95% CI)^a^ | p |
| YouTube Hours | 1.09 (1.03-1.14)* | 0.001 |
| Social Media Hours | 1.14 (1.09-1.20)* | < 0.001 |
| TV Hours | 1.11 (1.05-1.17)* | < 0.001 |
| Video Game Hours | 1.11 (1.05-1.16)* | < 0.001 |
| Browsing Web Hours | 1.16 (1.10-1.23)* | < 0.001 |
| Total Screen Time Hours | 1.04 (1.03-1.06)* | < 0.001 |
| **Social Media Platform Use** | PR (95% CI)^a^ | p |
| Facebook | 1.28 (1.09-1.51)* | 0.003 |
| Instagram | 1.34 (1.12-1.59)* | 0.001 |
| TikTok | 1.38 (1.17-1.61)* | < 0.001 |
| Twitter | 1.47 (1.22-1.76)* | < 0.001 |
| Snapchat | 1.22 (1.03-1.43)* | 0.019 |
| Twitch | 1.40 (1.14-1.72)* | 0.001 |
| Note: Each cell represents the abbreviated outputs of 12 modified Poisson regression models with screen time and social media platform use as the independent variables and weight-related bullying as the dependent variable. Preconstructed sample weighting applied to all analyses.  ***** indicates statistical significance (p < 0.05).  PR = Prevalence ratio; CI = Confidence interval  ^a^Adjusted for age, race/ethnicity, body mass index z-score classification, and family income adequacy. | | |
