# Supplementary material for: Screen time, social media use, and weight-related bullying victimization: Findings from an international sample of adolescents
Source: PLoS One. 2024 Apr 17;19(4):e0299830. doi: 10.1371/journal.pone.0299830 (PMC11023391; doi:10.1371/journal.pone.0299830)
Supplement: S3 Table — (DOCX) [file pone.0299830.s003.docx]

| S3 Table.  Associations between Screen Time and Social Media Platform Use and Weight-Related Bullying among Adolescent Participants in Australia from the 2020 International Food Policy Study (n = 1,595) | | |
| --- | --- | --- |
| **Screen Time, Hours per Weekday** | PR (95% CI)^a^ | p |
| YouTube Hours | 1.10 (1.04-1.16)* | 0.001 |
| Social Media Hours | 1.18 (1.11-1.25)* | < 0.001 |
| TV Hours | 1.09 (1.02-1.16)* | 0.007 |
| Video Game Hours | 1.14 (1.08-1.20)* | < 0.001 |
| Browsing Web Hours | 1.23 (1.16-1.31)* | < 0.001 |
| Total Screen Time Hours | 1.05 (1.03-1.06)* | < 0.001 |
| **Social Media Platform Use** | PR (95% CI)^a^ | p |
| Facebook | 1.83 (1.48-2.25)* | < 0.001 |
| Instagram | 1.48 (1.23-1.80)* | < 0.001 |
| TikTok | 1.26 (1.05-1.51)* | 0.012 |
| Twitter | 2.45 (2.05-2.93)* | < 0.001 |
| Snapchat | 1.16 (0.96-1.40) | 0.135 |
| Twitch | 1.87 (1.52-2.29)* | < 0.001 |
| Note: Each cell represents the abbreviated outputs of 12 modified Poisson regression models with screen time and social media platform use as the independent variables and weight-related bullying as the dependent variable. Preconstructed sample weighting applied to all analyses.  ***** indicates statistical significance (p < 0.05).  PR = Prevalence ratio; CI = Confidence interval  ^a^Adjusted for age, race/ethnicity, body mass index z-score classification, and family income adequacy. | | |
