# Supplementary material for: Screen time, social media use, and weight-related bullying victimization: Findings from an international sample of adolescents
Source: PLoS One. 2024 Apr 17;19(4):e0299830. doi: 10.1371/journal.pone.0299830 (PMC11023391; doi:10.1371/journal.pone.0299830)
Supplement: S4 Table — (DOCX) [file pone.0299830.s004.docx]

| S4 Table.  Associations between Screen Time and Social Media Platform Use and Weight-Related Bullying among Adolescent Participants in the United Kingdom from the 2020 International Food Policy Study (n = 1,521) | | |
| --- | --- | --- |
| **Screen Time, Hours per Weekday** | PR (95% CI)^a^ | p |
| YouTube Hours | 1.13 (1.04-1.23)* | 0.005 |
| Social Media Hours | 1.23 (1.14-1.32)* | < 0.001 |
| TV Hours | 1.07 (0.99-1.17) | 0.092 |
| Video Game Hours | 1.14 (1.06-1.24)* | 0.001 |
| Browsing Web Hours | 1.19 (1.07-1.32)* | 0.001 |
| Total Screen Time Hours | 1.05 (1.03-1.08)* | < 0.001 |
| **Social Media Platform Use** | PR (95% CI)^a^ | p |
| Facebook | 1.44 (1.12-1.86)* | 0.005 |
| Instagram | 1.47 (1.13-1.90)* | 0.004 |
| TikTok | 1.40 (1.10-1.79)* | 0.007 |
| Twitter | 1.32 (1.03-1.71)* | 0.031 |
| Snapchat | 1.49 (1.16-1.91)* | 0.002 |
| Twitch | 1.37 (0.98-1.93) | 0.065 |
| Note: Each cell represents the abbreviated outputs of 12 modified Poisson regression models with screen time and social media platform use as the independent variables and weight-related bullying as the dependent variable. Preconstructed sample weighting applied to all analyses.  * indicates statistical significance (p < 0.05).  PR = Prevalence ratio; CI = Confidence interval  ^a^Adjusted for age, race/ethnicity, body mass index z-score classification, and family income adequacy. | | |
