# Supplementary material for: Screen time, social media use, and weight-related bullying victimization: Findings from an international sample of adolescents
Source: PLoS One. 2024 Apr 17;19(4):e0299830. doi: 10.1371/journal.pone.0299830 (PMC11023391; doi:10.1371/journal.pone.0299830)
Supplement: S5 Table — (DOCX) [file pone.0299830.s005.docx]

| S5 Table.  Associations between Screen Time and Social Media Platform Use and Weight-Related Bullying among Adolescent Participants in the United States from the 2020 International Food Policy Study (n = 1,582) | | |
| --- | --- | --- |
| **Screen Time, Hours per Weekday** | PR (95% CI)^a^ | p |
| YouTube Hours | 1.07 (0.98-1.16) | 0.121 |
| Social Media Hours | 1.12 (1.04-1.21)* | 0.004 |
| TV Hours | 0.99 (0.91-1.08) | 0.823 |
| Video Game Hours | 1.05 (0.96-1.14) | 0.264 |
| Browsing Web Hours | 1.23 (1.14-1.34)* | < 0.001 |
| Total Screen Time Hours | 1.03 (1.01-1.05)* | 0.013 |
| **Social Media Platform Use** | PR (95% CI)^a^ | p |
| Facebook | 1.67 (1.26-2.22)* | < 0.001 |
| Instagram | 1.48 (1.11-1.98)* | 0.007 |
| TikTok | 0.96 (0.75-1.23) | 0.774 |
| Twitter | 1.77 (1.40-2.25)* | < 0.001 |
| Snapchat | 1.14 (0.89-1.48) | 0.281 |
| Twitch | 1.49 (1.13-1.96)* | 0.004 |
| Note: Each cell represents the abbreviated outputs of 12 modified Poisson regression models with screen time and social media platform use as the independent variables and weight-related bullying as the dependent variable. Preconstructed sample weighting applied to all analyses.  ***** indicates statistical significance (p < 0.05).  PR = Prevalence ratio; CI = Confidence interval  ^a^Adjusted for age, race/ethnicity, body mass index z-score classification, and family income adequacy. | | |
