# Supplementary material for: Screen time, social media use, and weight-related bullying victimization: Findings from an international sample of adolescents
Source: PLoS One. 2024 Apr 17;19(4):e0299830. doi: 10.1371/journal.pone.0299830 (PMC11023391; doi:10.1371/journal.pone.0299830)
Supplement: S6 Table — (DOCX) [file pone.0299830.s006.docx]

| S6 Table.  Associations between Screen Time and Social Media Platform Use and Weight-Related Bullying among Adolescent Participants in Mexico from the 2020 International Food Policy Study (n = 1,823) | | |
| --- | --- | --- |
| **Screen Time, Hours per Weekday** | PR (95% CI)^a^ | p |
| YouTube Hours | 1.05 (0.95-1.16) | 0.390 |
| Social Media Hours | 1.06 (0.97-1.17) | 0.204 |
| TV Hours | 0.95 (0.85-1.06) | 0.346 |
| Video Game Hours | 1.09 (1.00-1.20) | 0.056 |
| Browsing Web Hours | 1.05 (0.94-1.16) | 0.410 |
| Total Screen Time Hours | 1.02 (0.99-1.05) | 0.260 |
| **Social Media Platform Use** | PR (95% CI)^a^ | p |
| Facebook | 1.08 (0.74-1.59) | 0.679 |
| Instagram | 1.07 (0.80-1.43) | 0.639 |
| TikTok | 1.27 (0.96-1.69) | 0.098 |
| Twitter | 1.49 (1.13-1.98)* | 0.005 |
| Snapchat | 1.24 (0.93-1.65) | 0.138 |
| Twitch | 1.81 (1.33-2.46)* | < 0.001 |
| Note: Each cell represents the abbreviated outputs of 12 modified Poisson regression models with screen time and social media platform use as the independent variables and weight-related bullying as the dependent variable. Preconstructed sample weighting applied to all analyses.  * indicates statistical significance (p < 0.05).  PR = Prevalence ratio; CI = Confidence interval  ^a^Adjusted for age, race/ethnicity, body mass index z-score classification, and family income adequacy. | | |
