# Supplementary material for: Screen time, social media use, and weight-related bullying victimization: Findings from an international sample of adolescents
Source: PLoS One. 2024 Apr 17;19(4):e0299830. doi: 10.1371/journal.pone.0299830 (PMC11023391; doi:10.1371/journal.pone.0299830)
Supplement: S7 Table — (DOCX) [file pone.0299830.s007.docx]

| S7 Table.  Associations between Screen Time and Social Media Platform Use and Weight-Related Bullying among Adolescent Participants in Chile from the 2020 International Food Policy Study (n = 1,615) | | |
| --- | --- | --- |
| **Screen Time, Hours per Weekday** | PR (95% CI)^a^ | p |
| YouTube Hours | 1.04 (0.96-1.12) | 0.352 |
| Social Media Hours | 1.03 (0.95-1.11) | 0.480 |
| TV Hours | 1.05 (0.97-1.14) | 0.217 |
| Video Game Hours | 1.07 (0.99-1.14) | 0.081 |
| Browsing Web Hours | 0.96 (0.88-1.05) | 0.345 |
| Total Screen Time Hours | 1.01 (0.99-1.04) | 0.262 |
| **Social Media Platform Use** | PR (95% CI)^a^ | p |
| Facebook | 0.86 (0.69-1.09) | 0.216 |
| Instagram | 0.98 (0.74-1.31) | 0.904 |
| TikTok | 1.20 (0.94-1.53) | 0.146 |
| Twitter | 1.14 (0.87-1.51) | 0.334 |
| Snapchat | 1.39 (1.03-1.87)* | 0.033 |
| Twitch | 1.08 (0.80-1.44) | 0.627 |
| Note: Each cell represents the abbreviated outputs of 12 modified Poisson regression models with screen time and social media platform use as the independent variables and weight-related bullying as the dependent variable. Preconstructed sample weighting applied to all analyses.  * indicates statistical significance (p < 0.05).  PR = Prevalence ratio; CI = Confidence interval  ^a^Adjusted for age, race/ethnicity, body mass index z-score classification, and family income adequacy. | | |
